# Physician-guided deep learning model for assessing thymic epithelial tumor volume

**DOI:** 10.1117/1.JMI.12.4.046501

**Published:** 2025-08-13

**Authors:** Nirmal Choradia, Nathan Lay, Alex Chen, James Latanski, Meredith McAdams, Shannon Swift, Christine Feierabend, Testi Sherif, Susan Sansone, Laercio DaSilva, James L. Gulley, Arlene Sirajuddin, Stephanie Harmon, Arun Rajan, Baris Turkbey, Chen Zhao

**Affiliations:** a National Cancer Institute, Center for Cancer Research, Thoracic and Gastrointestinal Malignancies Branch, Bethesda, Maryland, United States; b National Institutes of Health, National Cancer Institute, Center for Cancer Research, Artificial Intelligence Resource, Bethesda, Maryland, United States; c National Institutes of Health, Clinical Center, Radiology and Imaging Sciences, Bethesda, Maryland, United States; d National Cancer Institute, Center for Cancer Research, Center for Immuno-Oncology, Bethesda, Maryland, United States

**Keywords:** deep learning, thymic epithelial tumor, volumetric measurement

## Abstract

**Purpose:**

The Response Evaluation Criteria in Solid Tumors (RECIST) relies solely on one-dimensional measurements to evaluate tumor response to treatments. However, thymic epithelial tumors (TETs), which frequently metastasize to the pleural cavity, exhibit a curvilinear morphology that complicates accurate measurement. To address this, we developed a physician-guided deep learning model and performed a retrospective study based on a patient cohort derived from clinical trials, aiming at efficient and reproducible volumetric assessments of TETs.

**Approach:**

We used 231 computed tomography scans comprising 572 TETs from 81 patients. Tumors within the scans were identified and manually outlined to develop a ground truth that was used to measure model performance. TETs were characterized by their general location within the chest cavity: lung parenchyma, pleura, or mediastinum. Model performance was quantified on an unseen test set of 61 scans by mask Dice similarity coefficient (DSC), tumor DSC, absolute volume difference, and relative volume difference.

**Results:**

We included 81 patients: 47 (58.0%) had thymic carcinoma; the remaining patients had thymoma B1, B2, B2/B3, or B3. The artificial intelligence (AI) model achieved an overall DSC of 0.77 per scan when provided with boxes surrounding the tumors as identified by physicians, corresponding to a mean absolute volume difference between the AI measurement and the ground truth of $16.1\;{\mathrm{cm}}^{3}$ and a mean relative volume difference of 22%.

**Conclusion:**

We have successfully developed a robust annotation workflow and AI segmentation model for analyzing advanced TETs. The model has been integrated into the Picture Archiving and Communication System alongside RECIST measurements to enhance outcome assessments for patients with metastatic TETs.

## 1 Introduction

Response Evaluation Criteria in Solid Tumors (RECIST) has been used to measure tumor response to cancer therapeutics since 2000.^1^ An updated version (RECIST version 1.1), first published in 2009, provides guidance on the selection and measurement of tumor lesions when assessing response.^2^ However, tumors are three-dimensional (3D), and numerous individuals and groups have noted the limitations of RECIST and have proposed disease- and treatment-specific modifications.^3^^–^^5^

Thymic epithelial tumors (TETs) are rare malignancies that are uniquely challenging to measure with RECIST. TETs arise from epithelial cells within the thymus, are located in the anterior mediastinum, and tend to metastasize to the pleura and pericardium.^6^ Pleural metastases, in particular, are difficult to measure accurately using RECIST because the single longest axis occasionally includes lung parenchyma due to their curvilinear morphology.^7^ To better account for this deficiency, the International Thymic Malignancy Interest Group (ITMIG) has proposed a modification to RECIST for TETs, stating that pleural lesions should be measured in “short-axis” diameters at “two locations at three separate levels” with the sum representing pleural tumor burden. This approach has shown similar results for progression-free survival when compared with standard RECIST measurement (5.5 months versus 7 months).^8^

Another shortcoming of RECIST is that the tumor measurements do not accurately reflect changes in tumor burden.^9^^–^^11^ In one assessment of tumor progression, investigators reported that only 31.999% of patients noted to have disease progression were labeled as having progression due to the growth of the target lesions.^12^ Other investigators have raised concerns about the validity of conclusions made using RECIST-based endpoints.^13^^,^^14^ The generalizations made by RECIST necessitate the use of ranges to define the change in tumor size. In addition, there is major inter- and intra-observer variability in radiological tumor assessment according to RECIST.^15^

3D tumor measurement could address some of the limitations of RECIST and provide more precise tumor assessment. To date, however, attempts to broadly apply previously derived algorithms have proven inadequate,^16^^,^^17^ and none of the current approaches to 3D tumor measurement have achieved clinical use.^9^^,^^16^^,^^18^ TETs present a valuable opportunity for 3D assessment because of their variety of thoracic lesions. Our group previously evaluated the clinical utility of volumetric assessment for TETs and demonstrated improved detection of progressive disease (PD) compared with RECIST. This evaluation compared three methods: computer tomography (CT)-based RECIST, modified RECIST for malignant pleural mesothelioma, which also presents with curvilinear pleural masses, and volumetric measurement following criteria described by Ak et al.^19^ The analysis identified a 22% discordance with RECIST and a 15% discordance with modified RECIST, detecting progressive disease an average of 72 days earlier than RECIST (p=0.016). Although this represents a significant advancement, the process remains highly time-intensive and continues to depend on the radiologist’s interpretation of volumetric measurements.^20^ More recently, deep learning has been successfully used to segment TETs and other challenging pleural lesions such as those found in patients with mesothelioma.^21^^–^^24^ Although the results of recent segmentation methods for TETs are promising, these were developed using only early-stage TETs, which are usually well-circumscribed and primarily mediastinal in location. Advanced stage TETs remain a challenging population for automated detection and volumetric assessment due to their rarity and high number of metastatic deposits with complex growth patterns mediastinum and pleura.^8^

We hypothesized that 3D measurement could provide more accurate and consistent results if used as an adjunct to current standards of radiological assessment specific to advanced TETs. To this end, we sought to create a physician-guided deep learning model using a segmentation/annotation approach to assess metastatic TETs from CT scans. Here, we describe the development of an AI model for automated segmentation of TET volume and evaluate, in this case, using ground truth segmentation and the AI-developed segmentation, the effectiveness of this model in the clinical setting through comparison with ITMIG-modified RECIST measurement for patients with advanced TETs participating in a clinical trial.

## 2 Methods

### 2.1 Patient Population and Imaging Data

For this study, CT scans and clinical data were extracted for patients with advanced and metastatic TETs enrolled across four different National Institutes of Health (NIH) institutional review board–approved clinical trials (i.e., NCT01306045, NCT05104736, NCT04417660, and NCT03076554). Patient demographics, including gender and racial information, are described in Table 1. CT scans obtained between February 2011 and April 2024 were included in this study. CT data were anonymized for research use via the Biomedical Translational Research Informatics System (BTRIS) at the NIH, which contains all electronic health record information for patients seen at the NIH, with the final cohort identifying 81 patients for inclusion. All scans performed on patients during the trial, including both contrast-enhanced and noncontrast scans, were collected for analysis (Table 5 in the Appendix).

**Table 1:** Clinical characteristics of patients included in model development.

Category	No. (%)
Total	81
Age, y (median)	56
Gender	
Male	40 (49.4)
Female	41 (50.6)
Race	
White	57 (70.4)
African American	12 (14.8)
Asian	9 (11.1)
Other	3 (3.7)
Ethnicity	
Not Hispanic or Latino	78 (96.3)
Hispanic or Latino	3 (3.7)
Subtype	
Thymoma B1	4 (4.9)
Thymoma B2	13 (16.0)
Thymoma B2/B3	5 (6.2)
Thymoma B3	11 (13.6)
Thymic carcinoma	47 (58.0)
Thymoma unspecified	1 (1.2)
Lesion types	
Total	572
Mediastinal	239
Pleural	269
Parenchymal	64

### 2.2 Data Annotation

**Fig. 1:**
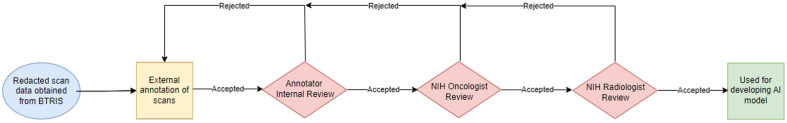
Methodological approach to scan annotation. AI, artificial intelligence; BTRIS, Biomedical Translational Research Informatics System; NIH, National Institutes of Health.

We took a physician-guided approach to identify tumor lesion regions: NC and CZ (thoracic medical oncologists) first assessed chest CT scans to identify tumor regions for annotation. At least two tumors (pleural metastases or mediastinal lesions), when available, were selected per scan with the goal of identifying tumors encompassing areas within and around the thoracic cavity. Volumetric contours of selected tumors were annotated manually by professional annotators by reviewing each image in the scan and using commercial software (V7 Labs) to outline previously identified tumors.^25^ These annotations underwent double verification, with the first review conducted by thoracic medical oncologists (NC and CZ), and if approved, a second review conducted by radiologists (JL and AS). Annotations rejected during either thoracic medical oncologist review or radiologist review were given comments and returned to the annotators for further adjustment and subsequent re-review (Fig. 1). These identified and outlined tumors were labeled as ground truth TETs.

### 2.3 Model Development

A 3D nnU-Net convolutional neural network architecture was used to build the TET segment model.^26^ nnU-Net is an off-the-shelf self-configuring variant of U-Net used for general medical image segmentation. It performs competitively on public challenge data sets and is used as the segmentation model for general medical imaging tools such as TotalSegmentator.^27^^–^^29^ Patients were randomly divided into a fivefold cross-validation or training set (61 patients) and a test set (20 patients) to avoid leakage bias, with all scans from an individual patient included in either training or testing sets.

**Fig. 2:**
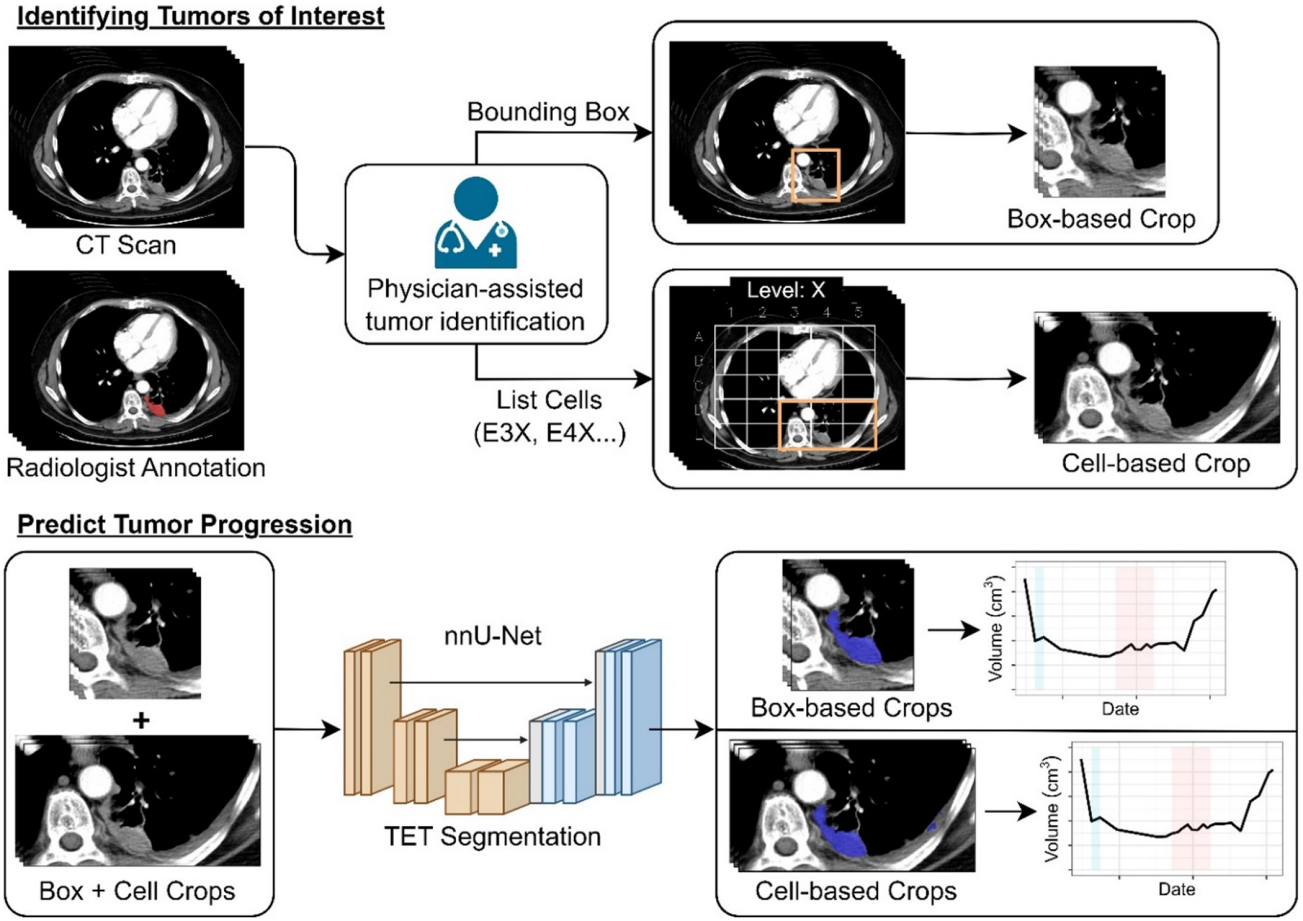
Different cropping strategies used to train the deep learning model. Demonstration of box-based crops versus cell-based crops in identifying tumors for volumetric assessment. CT, computed tomography; TET, thymic epithelial tumor.

Given the challenging appearance of TETs, the interest in tracking the growth of specific tumors, and the potential for false-positive segmentations when using the entire patient tumor burden, the nnU-Net was trained and evaluated on images cropped around metastatic TETs. During model development and evaluation, two cropping approaches were considered: (1) regions cropped to individual TETs using box-based crops drawn by physicians and (2) regions cropped based on prespecified regions of interest within the lung (cell-based crops), as illustrated in Fig. 2 and detailed below:

• In the box-based cropping method, we extracted a 3D region of interest surrounding the maximum boundaries of an individual TET. Therefore, each TET corresponded to one box for training. For clinical Picture Archiving and Communication System (PACS) interactivity, an entire box was considered.
• In the cell-based cropping method, we interactively selected crop regions that our PACS viewer does not support. A 5×5×5 grid was rendered on the CT scan around the lungs and was defined in terms of the extent of the lungs. For nnU-Net training, cells containing ground truth TET segmentations were utilized; therefore, more than one cell per TET could exist. At inference, the target grid cells that localized TETs of interest were then chosen and recorded in a database that was used for subsequent scans for the patient. The grid was always relative to the lungs and approximately retained correspondence across scans for the same patient due to the anatomical similarity of lungs across scans. Initial grid cell selection was performed manually by radiologists to ensure accurate inclusion of the tumor volume, rather than relying on automated proximity to a tumor centroid or boundary. These manually selected cells were stored and reused across longitudinal scans for consistent follow-up assessment. This approach balances practicality with accuracy in real-world PACS environments where precise bounding box tools may be unavailable.
• Finally, a model trained from combined box-based and cell-based methods was considered.

All models were trained for 1000 epochs using the default 3D high-resolution nnU-Net configuration. The model was trained as a binary segmentation task, with voxels classified as either tumor or background. Training loss (cross-entropy + Dice) was monitored throughout model development. The best model for each implementation was selected based on the best Dice similarity coefficient (DSC) in the validation set during training.

In all training configurations, including the cell-based-only model, nnU-Net’s built-in data augmentation pipeline was applied. This included spatial transformations, intensity augmentations, and noise injection, among other techniques. These augmentations were used consistently across all models to enhance generalizability and mitigate overfitting, although their application alone did not close the performance gap between cell-based and box-based models.

### 2.4 Automated TET Location Classification

Using a stepwise approach, ground truth TETs were automatically classified as pleural, mediastinal (primary lesion and nodes), or lung parenchymal. First, lung segmentation was performed using TotalSegmentator.^29^ Quartiles of coordinates of the lung segmentation were calculated and included the 25 and 75 percentiles of X and Y coordinates of the lung segmentation voxels. Any TET that did not border the lung segmentation boundary was simply labeled as a parenchymal lesion. Any TET that met this criterion but was bordering the segmentation boundary and had a bounding box center $\left(X_{t},Y_{t},Z_{t}\right)$ satisfying both $X_{25\%}\leq X_{t}\leq X_{75\%}$ and $Y_{75\%}\geq Y_{t}$ was labeled as mediastinal. All other TETs not classified by these rules were labeled as pleural.

### 2.5 Proof-of-Concept Clinical Evaluation

The model was used to evaluate scans for patients with TETs that were not included in model development. AI prediction of tumor size was measured against the overall RECIST measurement of thoracic tumors. RECIST measurements for these scans were previously assessed (by CZ, MM, and AR) for the clinical trials. These values were compared with size cutoffs for progression and response per RECIST criteria.

### 2.6 Statistical Analysis

The nnU-Net test performance was quantified in four ways: scan DSC, individual tumor DSC, absolute volume difference (AVD), and relative volume difference (RVD). These four statistics were calculated on box-based cropped images and cell-based cropped images, giving a total of 8 statistics. Statistical significance was assessed using a two-sided Mann–Whitney–Wilcoxon test between performance on cell- and box-based crops. Scan DSC was calculated directly on the ground truth segmentation mask and inference segmentation where all tumors were simultaneously treated as foreground. In contrast to scan DSC, tumor DSC was calculated per ground truth tumor and averaged over all individual tumors. Per-crop DSC, on the other hand, refers to the average DSC across all 3D cropped input regions (each of which may contain multiple tumors or portions of tumors), treating each cropped volume as a separate evaluation unit. Although both tumor DSC and per-crop DSC measure segmentation accuracy using the Dice coefficient, they differ in how the segmentation masks are grouped and averaged. This distinction is important for interpreting performance in scenarios with multiple tumors per region.

DSC quantifies segmentation accuracy, producing scores ranging between 0 (completely different) and 1 (identical). Mathematically, the DSC is defined as

$$\mathrm{DSC}(G,S)=\frac{2|G\cap S|}{\left|G|+|S\right|},$$

where G is the ground truth segmentation mask, S is the inference segmentation mask, |G| and |S| count the number of TET voxels in G and S, and |G∩S| counts the number of TET voxels in common between G and S.

Although DSC measures the accuracy of segmentation, AVD and RVD measure the accuracy of volume measurement, which is pertinent for the clinical application of this work. These are given as follows:

AVD(G,S)=v||G|−|S||,

$$RVD(G,S)=\frac{\left||G|-|S|\right|}{\left|G\right|},$$

where v is the volume of a single voxel in ${\mathrm{cm}}^{3}$ and |G| and |S| are the counts of TET voxels in segmentation masks G and S.

Parenchymal, mediastinal, and pleural performance of the model were calculated on a per-scan and per-tumor basis. This was done by matching overlapping inference mask-connected components with ground truth TETs and then calculating DSC, AVD, and RVD for each ground truth TET. Consequently, false-positive connected components are ignored for location-based TET performance. This contrasts with the overall performance, which is evaluated on inference and segmentation masks for an entire crop or scan.

## 3 Results

For the development of the AI model, 231 scans from 81 patients were annotated and approved for evaluation. Of all TET patients, 47 (58.0%) had thymic carcinoma; the remaining patients had type B thymomas: B1, B2, B2/B3, and B3. A total of 572 tumors were used, with the majority located in the mediastinum (n=239) or the pleura (n=269), and a few located in the lung parenchyma (n=64). Further demographic data are detailed in Table 1. The 572 tumors were split into a training set containing 431 tumors (pleura = 198, mediastinum = 182, parenchyma = 51) from 170 scans for 61 patients and a test set with 141 tumors (pleura = 71, mediastinum = 57, parenchyma = 13) from 61 scans for 20 patients. Both sets had approximately the same proportion of tumors at each location as the entire cohort (Table 6 in the Appendix).

We found a per-crop DSC of 0.63 on cell-based crops and a per-crop DSC of 0.80 on the model trained on the combined cell-based and box-based crops (Table 2). In addition, the performance evaluated on box-based crops was markedly better than on cell-based crops (per-crop p-value = 1.7e-−4; per-scan p-value = 2.1e−3). Therefore, we employed the combined model as this gave the most accurate approach and focused our downstream analysis on box-based cropped regions of interest.

**Table 2:** Model performance based on cropping strategies.

Training images	Per-crop DSC (*SD*)			Per-scan DSC (*SD*)		
	Cell	Box	p-Value	Cell	Box	p-Value
Cell-based crops	0.54 (0.30)	0.59 (0.32)	0.23	0.49 (0.30)	0.64 (0.24)	0.023
Box-based crops	0.49 (0.33)	0.78 (0.17)	1.4e−8	0.49 (0.31)	0.79 (0.11)	9.4e−7
Cell-based + box-based crops	0.63 (0.31)	0.80 (0.16)	1.7e−4	0.59 (0.31)	0.77 (0.18)	2.1e−3

Cell-based and box-based cropping DSC performance of 3D nnU-Net when trained on cell-based crops only, box-based crops only, and combined cell-based and box-based crops. Evaluated on the held-out test set (N=61 scans) for box-based and cell-based crops. p-Values were calculated using a two-sided Mann–Whitney–Wilcoxon test between DSC performance on cell- and box-based crops. DSC, Dice similarity coefficient.

**Fig. 3:**
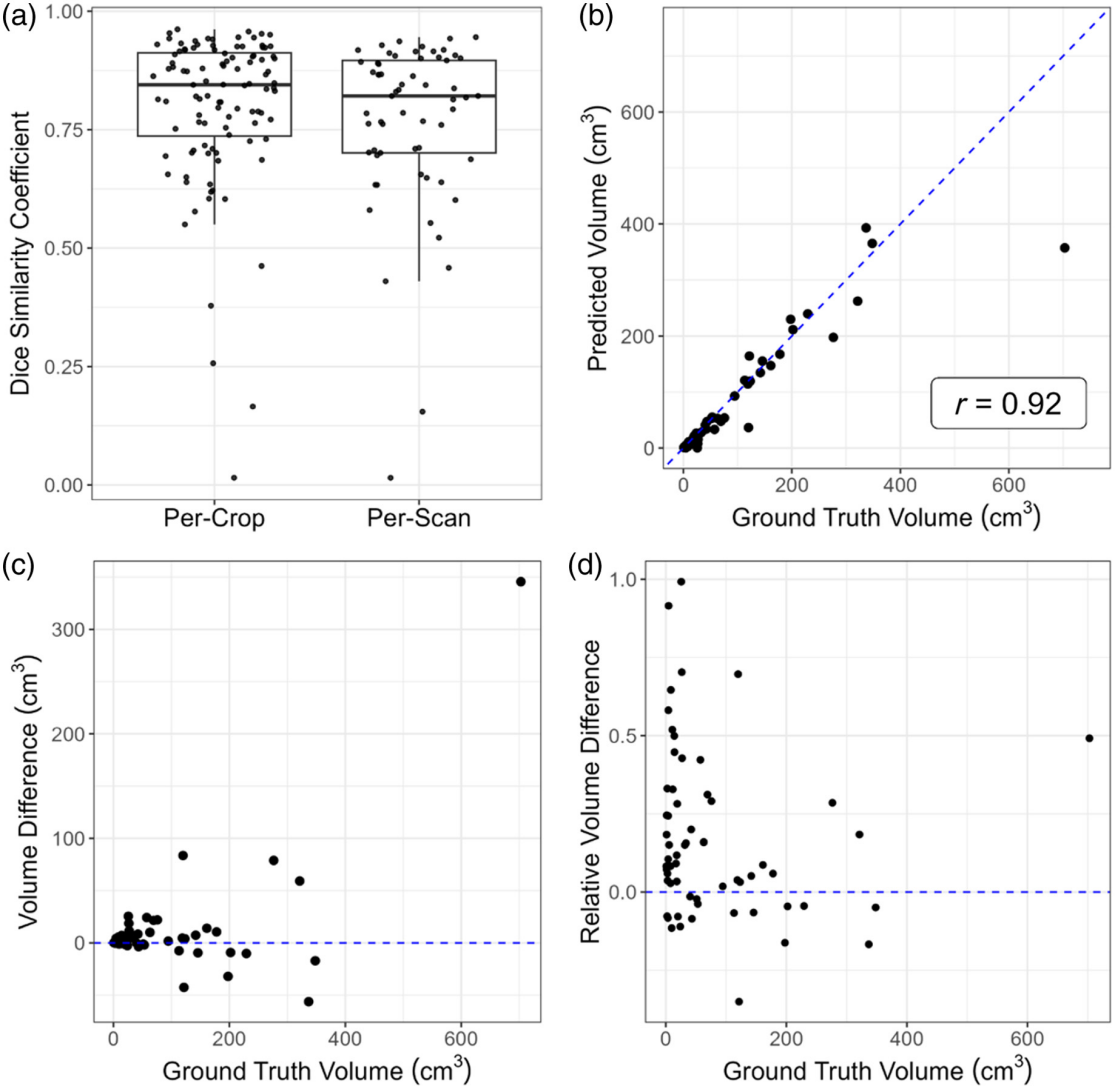
Deep learning model performance. (a) Quantitative performance measured by the Dice similarity coefficient (DSC) at a per-crop and per-scan level. (b) Regression of AI-predicted volume (${\mathrm{cm}}^{3}$) versus ground truth volume (${\mathrm{cm}}^{3}$). (c) Regression of absolute volume difference (ground truth volume−predicted volume) versus ground truth volume (${\mathrm{cm}}^{3}$) and (d) relative volume difference $\left(\frac{\text{ground truth volume}-\text{predicted volume}}{\text{ground truth volume}}\right)$ versus ground truth volume (${\mathrm{cm}}^{3}$) evaluated on the held-out test set (N=61 scans) for box-based crops at a per-scan level.

Box plots showing the DSC distribution for the test set at both the per-scan level and the per-crop level for cell-based crops and box-based crops are shown in Fig. 3(a). The model additionally had a higher overall DSC of 0.77 per scan and of 0.80 per individual tumor when evaluating box-based crops. Based on location, we obtained DSCs of 0.87 for identifying lung parenchymal tumors, 0.81 for mediastinal tumors, and 0.73 for pleural tumors. By contrast, cell-based crops led to lower DSCs of 0.59 per scan and 0.63 per crop. The corresponding DSC values by location were 0.74 for the lung parenchyma, 0.79 for the mediastinum, and 0.67 for the pleura (Table 3).

**Table 3:** Model performance based on lesion locations.

Structure	Per-crop DSC mean (*SD*)		Per-scan DSC	
	Cell	Box	Cell	Box
Overall	0.63 (0.31)	0.80 (0.16)	0.59 (0.31)	0.77 (0.18)
Lung parenchyma	0.74 (0.30)	0.87 (0.08)	N/A	N/A
Mediastinum	0.79 (0.22)	0.81 (0.20)	N/A	N/A
Pleura	0.67 (0.28)	0.73 (0.23)	N/A	N/A

Mean DSCs for parenchyma, mediastinum, and pleura TETs with the use of the cell-based and box-based cropping strategies. There is no per-scan DSC for specific TET types because of ambiguities with false-positive segmentations. DSC, Dice similarity coefficient; TET, thymic epithelial tumor.

AVD and RVD were calculated across the test set for both approaches. The box-based approach overall did better than the cell-based approach in the parenchyma and mediastinum with lower AVD and RVD. This was reversed with pleural lesions, for which the cell-based approach proved more accurate (Table 4). The relation between predicted volume versus ground truth volume was further evaluated by a correlation analysis, which showed an r=0.92 [Fig. 3(b)]. However, there were some notable differences between what was achieved by the AI model and what was considered ground truth according to the AVD and RVD [Figs. 3(c) and 3(d)].

**Table 4:** Volume differences based on lesion locations.

Structure	Absolute volume difference mean (*SD*)		Relative volume difference mean (*SD*)	
	Cell	Box	Cell	Box
Overall	46.3	16.1	1.85	0.22
Lung parenchyma	48.06 (135.10)	0.44 (0.45)	15.59 (42.84)	0.17 (0.15)
Mediastinum	15.41 (30.44)	10.98 (23.04)	744.76 (3305.96)	526.05 (2516.73)
Pleura	12.34 (25.12)	13.63 (31.89)	2.52 (6.10)	64.21 (477.67)

Absolute volume difference (${\mathrm{cm}}^{3}$) and relative volume difference overall and for parenchyma, mediastinum, and pleura thymic epithelial tumors with use of the cell-based and box-based cropping strategies.

**Fig. 4:**
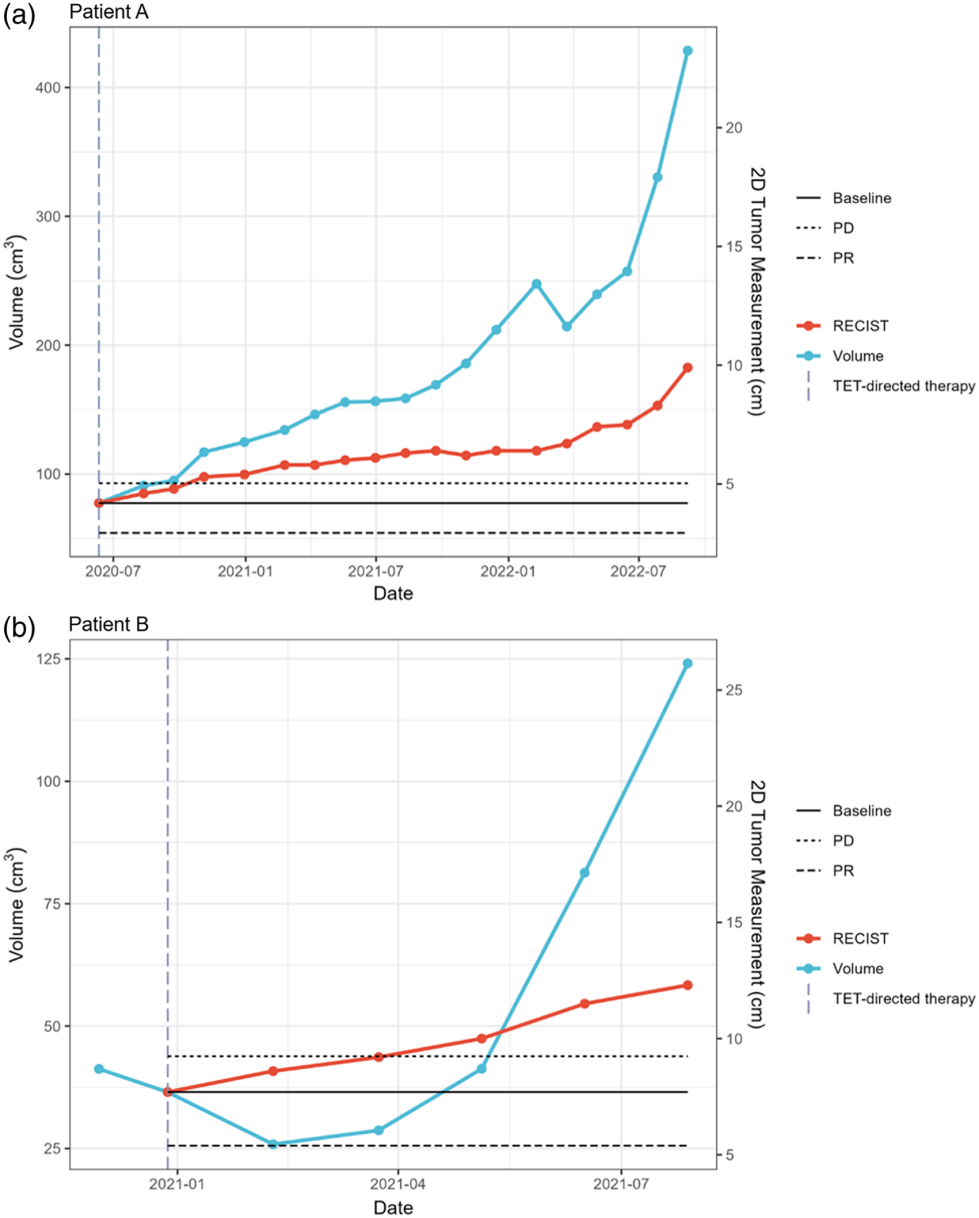
Longitudinal assessment of tumor volume with use of the deep learning model compared with RECIST measurements. Serial volumetric and RECIST assessment for two patients with metastatic TETs receiving immunotherapy. The vertical dashed line marks the start of treatment. (a) Tumor measurement of TET patient A; (b) Tumor measurement of TET patient B. PD, progressive disease; PR, partial response; RECIST, Response Evaluation Criteria in Solid Tumors; TET, thymic epithelial tumor.

**Fig. 5:**
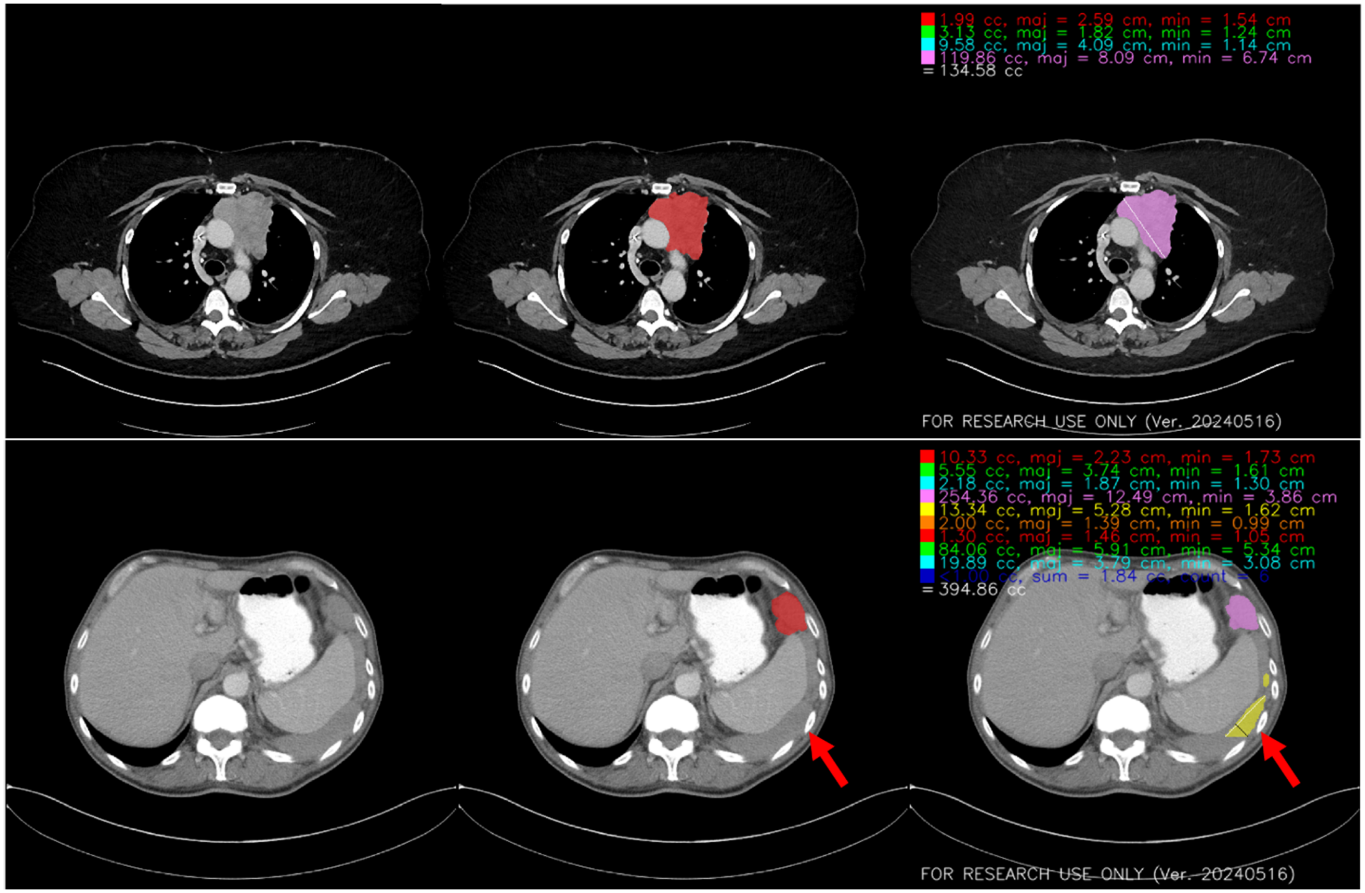
Examples of CT image, ground truth segmentation, and box-based cropping inference segmentation. The top row shows a good inference segmentation (per-scan DSC of 0.92), and the bottom row shows a red arrow pointing to a false-positive segmentation on fluid (per-scan DSC of 0.55). From left to right: CT image shown with mediastinum windowing, ground truth segmentation (red) overlaid on the CT image, and inference segmentation overlaid on the CT image. White lines are major axis measurements (maj) and black lines are minor axis measurements (min). CT; computed tomography; DSC, Dice similarity coefficient.

**Fig. 6:**
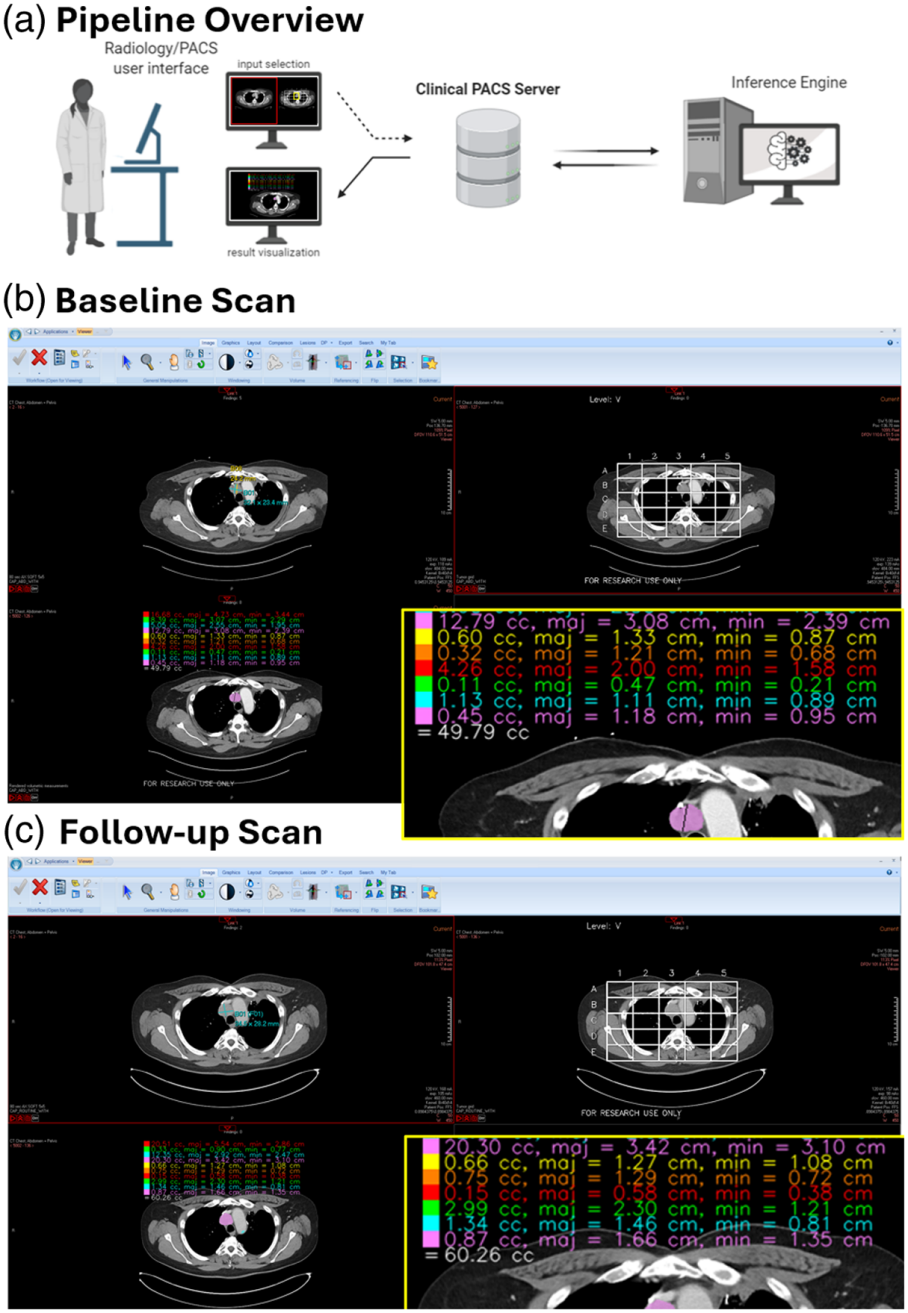
Proof-of-concept PACS implementation at our institution using Philips Carestream. (a) Diagram demonstrating dataflow from PACS to the inference engine. (b) In a representative patient, cell VB3 was selected for tumor segmentation. The tumor is automatically segmented and volumetric outputs as well as Response Evaluation Criteria in Solid Tumors (RECIST)-relevant measurements are reported. (c) In a follow-up scan, this lesion is automatically identified and tracked using the same selection input. The physician can then report the volumetric increase from 12.79 to 20.30 cc between scans. In both scans, the original CT series with radiologist-defined 2D measurements [top left panels (b) and (c)], the grid assignment [top right panels (b) and (c)], and the deep learning outputs [bottom left panels (b) and (c)] are available.

The AI model was used for longitudinal assessment of tumor volume compared with RECIST. Examples of these longitudinal measurements are shown for two patients in Fig. 4. Of note, in our clinical trials of immunotherapy for recurrent TETs, participants could continue to receive protocol-directed therapy after radiological progression per RECIST version 1.1 if previously defined clinical criteria were met. In one patient, a notable decrease in tumor burden after treatment can be seen in the first scan by volumetric measurement compared with RECIST measurement. Two visual examples of CT images, ground truth segmentations, and inference segmentations with TET measurements as seen on PACS are presented in Figs. 5 and 6 in the Appendix.

## 4 Discussion

RECIST was created through a joint effort of the World Health Organization, the National Cancer Institutes of the United States and Canada, and the European Organization for Research and Treatment of Cancer in 2000 as a standardized measurement for evaluating tumors.^1^ RECIST was based on a retrospective statistical evaluation of measurements from eight pharmaceutical-sponsored clinical trials involving 569 patients.^30^ However, RECIST does not account for the 3D nature of tumors because measurements are performed on 2D axial sections. Furthermore, modifications to RECIST have been proposed in cancers with difficult anatomical presentation and spread, such as TETs.^8^ In the current study, we evaluated the feasibility of using a deep-learning–based AI approach with a novel algorithm to quantify volumes of intrathoracic TETs. Unlike that of more common lung cancers, the thoracic distribution of metastatic TETs tends to be more pleural and mediastinal in nature. Given the unique and challenging clinical presentation of advanced TET, we have designed a method that utilizes a physician-guided approach to segment tumors of interest.

Different from standard medical imaging annotation workflows, which often rely on a single annotator or lack iterative physician involvement, our multitiered protocol ensured high clinical fidelity by involving domain experts at each stage. This was particularly important for TETs, where the precise anatomical and pathological context is critical due to their rarity and complexity. The sequential review by both oncologists and radiologists reflects a rigorous quality control mechanism, minimizing annotation noise and improving downstream model robustness. Furthermore, by leveraging existing PACS infrastructure and incorporating noninteractive annotation tools, our approach provides a practical and scalable solution suitable for real-world clinical deployment where traditional bounding-box or interactive annotations are often infeasible.

Previous studies have used the DSC as a metric of goodness-of-fit across a range of tumors with widely varying values based on tumor site.^31^ For thoracic cancer models, the DSC has generally been found to be between 0.68 and 0.78, depending on the approach, areas assessed, and type of scan used.^24^^,^^32^^–^^34^ These models generally suffer in the case of tumors located in the mediastinum or along the pleura because of the similar density of the surrounding tissue.^24^^,^^32^ It is noteworthy that our model achieved an overall DSC of 0.77 in per-scan box crops for primarily pleural and mediastinal-based tumors. This result illustrates the ability of our model to override many of the difficulties faced by previous AI models.

We noted that the model performed significantly better on box-based crops than on cell-based crops, which could be attributed to the inferior precision of static grids. These findings emphasize the important role of physicians in identifying regions of interest for tumor identification. The combined model outperformed both the box-based and the cell-based models on their own. As a data augmentation process, the combined model is trained with more images than either the cell-based or the box-based model because the combined model uses images identified by both methods. This might partly explain the improved performance over models dedicated to specific crop types. As not all tumors were annotated on the CT scans, the cell-based model may overlap with unannotated TETs, which might explain the poorer performance of this model. However, it could be that the combination of the two approaches highlights the benefits of each. The box-based crops capture the tumor more precisely, whereas the cell-based crops include negative anatomical structures, which decreases false-positive segmentation.

Although the per-crop and per-scan DSCs appear similar between the box-only and combined models on box-based images, including cell-based crops during training introduced additional contextual variation and anatomical background, enriching the model’s exposure to both positive and negative samples. This enrichment may enhance the model’s ability to generalize and reduce false positives, particularly during inference on complex cases. Importantly, the incorporation of cell-based data was also motivated by institutional PACS viewer constraints, where bounding box input is not always supported. As such, this flexibility in training aligns with the goal of developing a clinically viable and versatile system adaptable to different operational environments.

Although our results focus on cropped regions of interest, we acknowledge that evaluating model performance on full CT volumes could provide a more comprehensive assessment of segmentation accuracy and false positive rates. However, our study was specifically designed to emulate clinical RECIST workflows, which center on lesion-specific tracking rather than whole-scan detection. As such, whole-volume annotations were not available, and evaluating performance across entire scans was beyond the intended scope of this work. We recognize this as a limitation and note that such analyses could be pursued in future studies to assess full-scan generalization and background discrimination.

In addition, our automated TET location classification relied on lung boundaries generated by TotalSegmentator to define anatomical grid regions. Although this segmentation tool has demonstrated strong performance in benchmark datasets, we were unable to quantitatively assess lung segmentation accuracy in our own dataset due to the absence of ground truth lung masks. As a result, we acknowledge that potential segmentation inaccuracies, such as partial lung coverage or boundary misalignments, could affect the anatomical categorization of tumors into pleural, mediastinal, or parenchymal regions. Although the location rules were designed to be robust to small deviations, this reliance on automated segmentation introduces a possible source of error and represents a limitation of the current classification framework.

The AI model developed here can improve tumor measurement when used in conjunction with RECIST. This can be seen with the two examples we have provided of tumor growth over time, as well as the correlation of AI-calculated tumor size with what is deemed to be the ground truth, for which we note the exceedingly high regression coefficient. Although formal evaluation of the time necessary for tumor volume assessment has not been done, the calculations are generally completed within 10 min of submission of the scan for evaluation. The speed at which the AI evaluation is done will allow evaluation alongside the RECIST calculation of tumors. Given the physician-guided nature of the AI approach, the model can be guided to the evaluation of only target-specific lesions and may be able to identify both response and progression sooner and more accurately than RECIST. The volumetric measurement of tumors both addresses issues with curvilinear lesions and provides a rapid assessment of tumor volume for comparison with previous scans. We have created this AI model with an eye toward future real-world use by ensuring that it can evaluate scans in a timely manner to aid physicians in assessing tumor volume.

Although the model was developed to allow clinicians to track and measure specific TETs via user-provided bounding boxes, some clinical environments have limited PACS image viewers that cannot accept bounding box input. Our system addresses this possible clinical limitation through its optional use of the 5×5×5 grid described earlier. A database stores a list of lung grid cells for cell-based cropping and inference with the AI, and this avoids the need for user-provided bounding boxes. For new patients not included in the database, the system initially generates a rendering of the lung grid overlaid on the CT scan. The radiologist then picks the lung grid cells that correspond with TETs to list in the database. This process is visually depicted in Fig. 6(a) in the Appendix, and we show examples of this system used with our institute’s PACS viewer in Figs. 6(b) and 6(c) in the Appendix.

Despite the advances mentioned above, our AI model also has some limitations. The first limitation is that the growth of the tumor beyond the identified bounding box may result in misrepresentation of tumor growth. Plans to expand this model include automatic sensing and growth of the bounding box to account for tumor growth. Another limitation is that the model may have difficulty in recognizing tumor versus mediastinal structures in noncontrast images, leading to erroneous volume measurements. Although we included several noncontrasted scans in our evaluation, we are exploring this limitation further using lung mask suppression to identify and exclude cardiac structures. Furthermore, this study was conducted at a single institute. Although TETs are rare diseases, a larger cohort at multiple institutions would be helpful in further validating our findings. In addition, a reader study could evaluate the effectiveness of the AI approach in relation to radiologists’ readings to evaluate the efficiency and accuracy of the model when used as an adjunct to TET evaluation. Finally, although we focused on nnU-Net due to its demonstrated superiority on our dataset, we recognize that validating the physician-guided annotation framework across a broader set of segmentation models (e.g., 3D TransUNet, V-Net variants, Transformer-based models) would further establish the robustness and adaptability of our approach. Formal benchmarking and cross-model evaluation remain an area of future work as resources permit.

Future work will include a reader study to thoroughly evaluate how the model can best be used by radiologists. In addition, we aim to expand the framework described in this study to other thoracic tumors with the understanding that each tumor type has intricacies that may require model adjustments.

## 5 Conclusion

To our knowledge, this is the first 3D physician-guided AI model to measure TET growth that can be successfully incorporated into a clinical workflow. Our model performed well in assessing volumetric growth trends and provides a more accurate assessment of changes in curvilinear pleural metastases. Similar studies can contribute to expanding and improving the current state of AI evaluation for thoracic tumors.

## 6 Appendix: Supplementary Material

Figure 6 illustrates the implementation of our analysis pipeline in a real-world scenario.

Table 5 summarizes the CT scan acquisition parameters used in this study, and Table 6 presents the demographic and clinical characteristics of the patients included.

**Table 5:** CT acquisition details.

Summary	Siemens	GE medical systems	Toshiba	Philips	Canon medical systems
# scans	250	10	3	11	7
XY spacing	0.826 (0.625 − 0.977)	0.781 (0.703 − 0.898)	0.820 (0.782 − 0.858)	0.820 (0.683 − 0.977)	0.858 (0.782 − 0.938)
Slice thickness	2 (1 − 5)	5 (5 − 5)	5 (5 − 5)	2 (2 − 5)	5 (5 − 5)
Contrast use	112 (45%)	4 (40%)	2 (67%)	11 (100%)	7 (100%)

All values are denoted as median (range) unless otherwise specified.

**Table 6:** Division of patients, scans, and lesions between the test set and the training set.

	Training set	Test set
TET subtype		
Total patients	61	20
Thymoma B1	4	0
Thymoma B2	11	2
Thymoma B2/B3	5	0
Thymoma B3	7	4
Thymic carcinoma	33	14
Thymoma unspecified	1	0
Scans	170	61
Lesion types		
Total	431	141
Mediastinal	182	71
Pleural	198	57
Lung parenchymal	51	13

## Data Availability

CT scans and annotation data can be found via https://github.com/NIH-MIP/CT_thymoma.
